# A Case of Stercoral Colitis Complicated by Ischemic Colitis in a Young Patient

**DOI:** 10.7759/cureus.26050

**Published:** 2022-06-17

**Authors:** Ese Uwagbale, Vimal Bodiwala, Solomon Agbroko, Elliot Bigajer

**Affiliations:** 1 Internal Medicine, Brookdale University Hospital Medical Center, New York, USA; 2 Gastroenterology and Hepatology, State University of New York Downstate Medical Center, New York, USA; 3 Obstetrics and Gynecology, Aspirus Keweenaw Hospital, Laurium, USA; 4 Obstetrics and Gynecology, Maimonides Medical Center, New York, USA; 5 Gastroenterology and Hepatology, Brookdale University Hospital Medical Center, New York, USA

**Keywords:** chronic constipation, constipation, fecal impaction, ischemic colitis, stercoral colitis

## Abstract

Stercoral colitis complicated by ischemic colitis is rare. Current literature has focused on the radiographic characteristics of stercoral colitis and management of bowel perforation resulting from complicated stercoral colitis. This case report describes possible challenges in diagnosing and managing stercoral colitis complicated by ischemic colitis. We present a case of stercoral colitis complicated by ischemic colitis in a 28-year-old woman who presented with lower gastrointestinal bleeding.

## Introduction

Stercoral colitis is inflammatory colitis with a mortality rate of 35% when associated with ischemic colitis [1]. In addition, stercoral colitis complicated by colonic perforation can lead to septic shock and a mortality rate as high as 60% if an accurate diagnosis is not made promptly [2].

Stercoral colitis is caused by fecal impaction leading to increased colonic intraluminal pressure. Complications of stercoral colitis include ischemic necrosis, ulcer formation, and colonic perforation. Stercoral colitis is frequently seen in older patients with chronic constipation, bed-bound patients, and sometimes younger patients with psychiatric conditions [1]. Patients with stercoral colitis can present with abdominal pain or vague symptoms and can sometimes be asymptomatic. 

## Case presentation

A 28-year-old woman with a past medical history of autism, seizure disorder, and chronic constipation presented to the emergency room with a one-day history of bright red blood per rectum with associated blood clots. The patient's mother reported a one-week history of constipation and over-the-counter laxative use before presenting to the emergency room. At baseline, the patient ambulates without assistance. Initial vitals showed a blood pressure of 96/67mmHg, pulse rate of 123 beats/minute, respiratory rate of 12 breaths/minute, and oxygen saturation of 97% on room air. Initial labs were significant for hemoglobin of 16.1g/dl, platelet count of 171,000, prothrombin time of 13, an international normalized ratio of 1.1, partial thromboplastin time of 26.2, blood urea nitrogen of 14, serum creatinine of 1.46, lactic acidosis with serum lactate of 6.1 and white blood cell (WBC) count of 15.3. The patient had abdominal distention without guarding or tenderness; rectal examination showed hard stool mixed with bright red blood.

**Figure 1:**
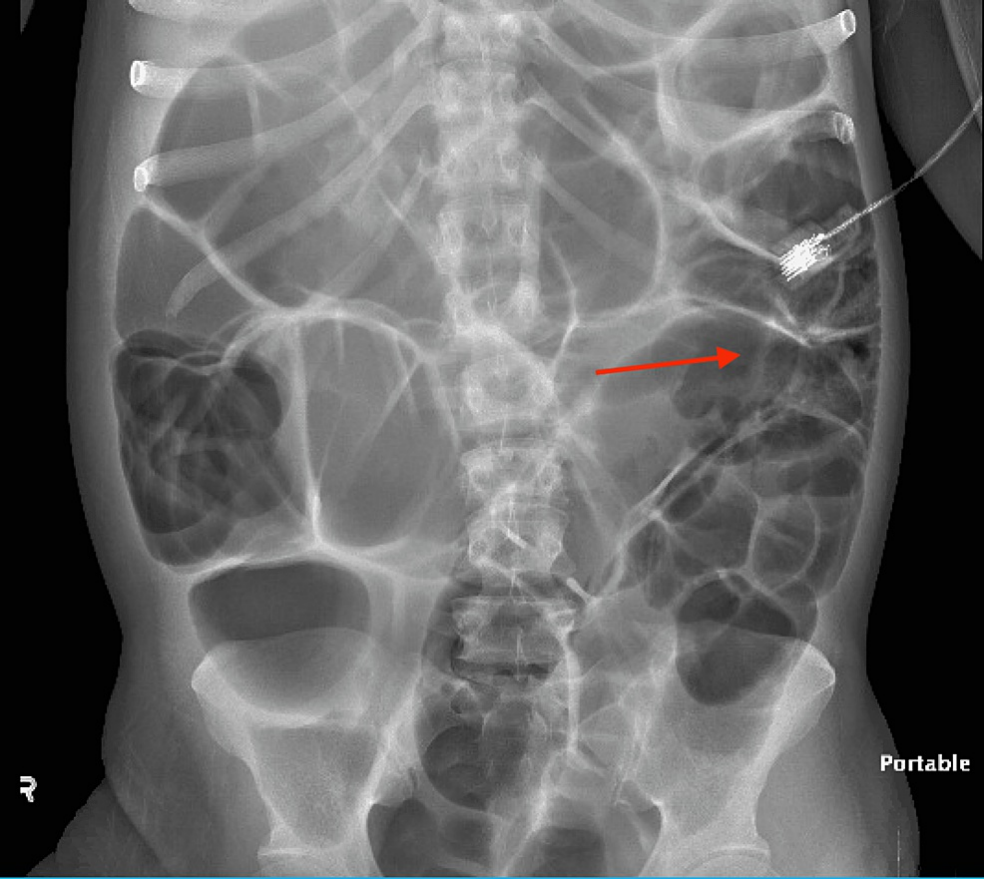
Abdominal X-ray with red arrow showing diffuse large and small bowel gas most suggestive of ileus

**Figure 2:**
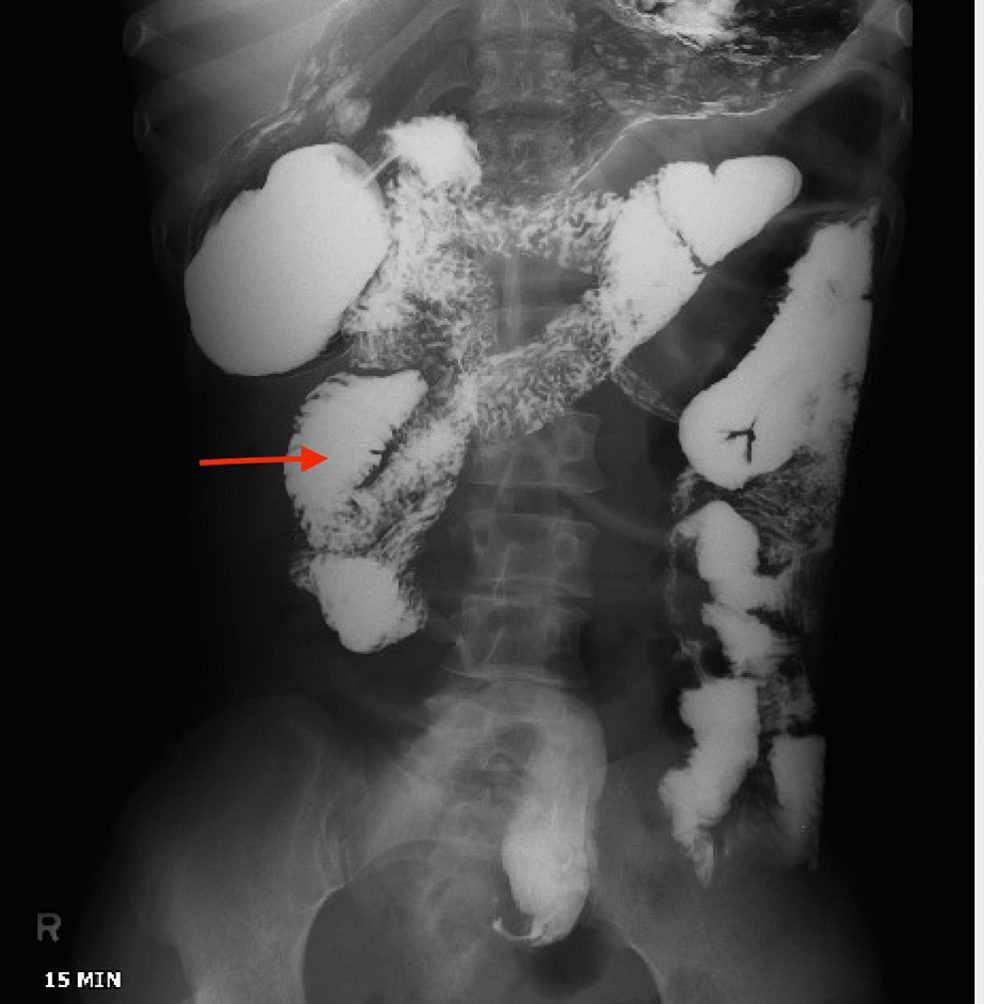
Small bowel study with red arrow showing persistent focal dilation of small bowel loops

Abdominal X-ray showed diffuse large and small bowel gas most suggestive of ileus without obstruction (Figure 1). A small bowel study was done, which showed persistent focal dilation of small bowel loops in the right upper quadrant and right mid-abdomen (Figure 2).

**Figure 3:**
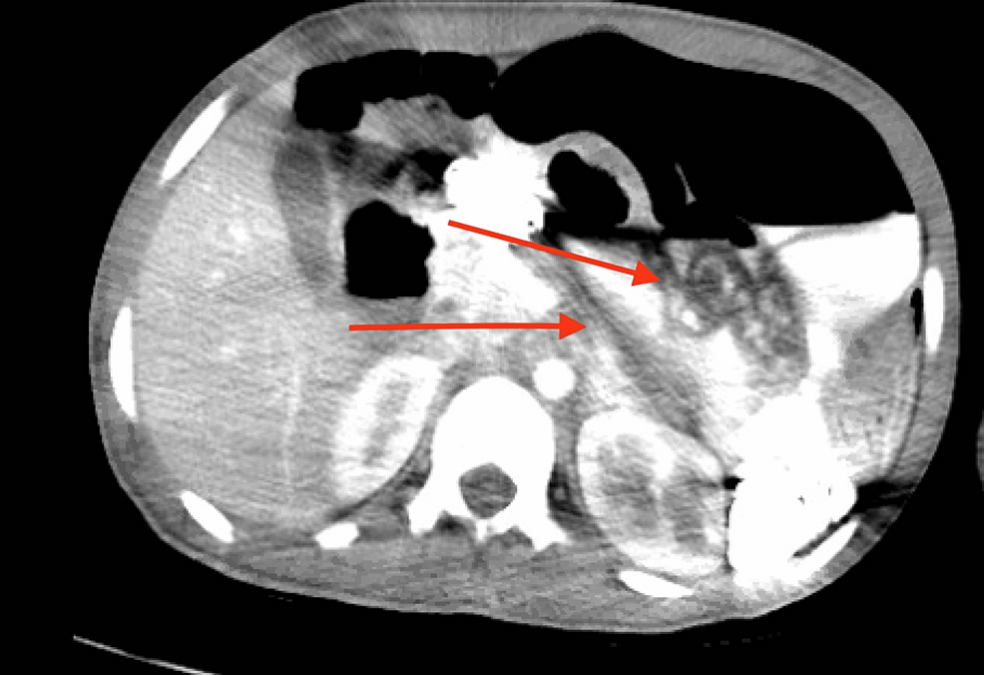
A computerized tomography scan of the abdomen with intravenous and oral contrast limited by significant streaking artifacts from inspissated and thick oral contrast in the gastrointestinal tract, as shown by the red arrows

A computerized tomography scan of the abdomen with intravenous and oral contrast (Figure 3) showed a significant stool burden, diffuse small and large bowel dilatation suggestive of ileus without abdominal obstruction; the study was limited by significant streaking artifacts from inspissated and thick oral contrast in the gastrointestinal tract.

**Figure 4:**
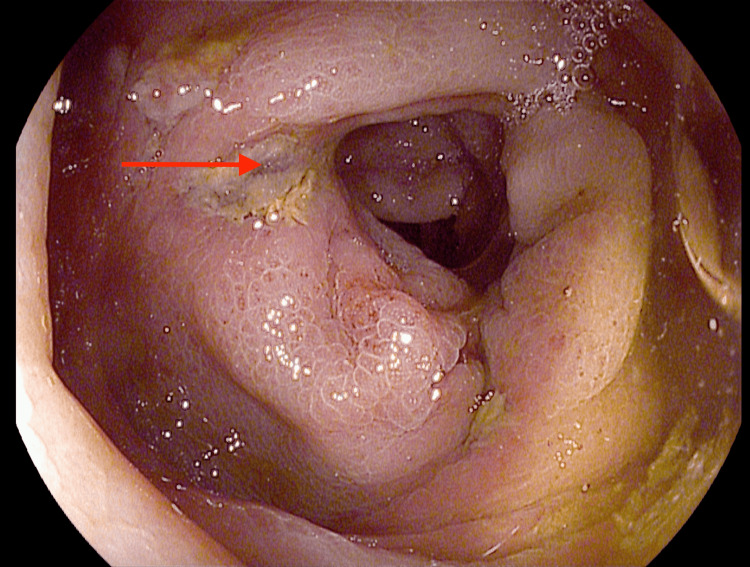
Red arrow showing linear ulceration in the descending colon

**Figure 5:**
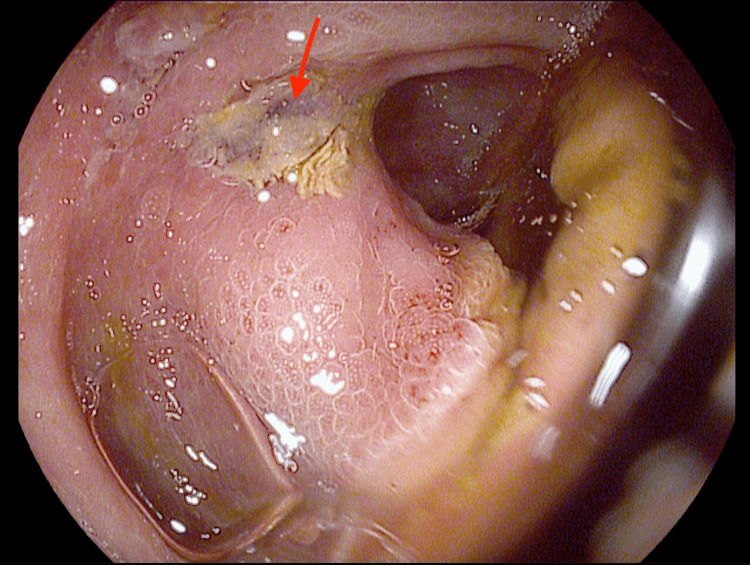
Red arrow showing linear ulceration in the descending colon

The patient was started on intravenous fluid hydration. Manual disimpaction was performed, and laxatives were started. The patient had a diagnostic colonoscopy that showed two abnormal erythematous, congested mucosa areas with linear ulceration in the descending colon (Figures 4-5), which was biopsied.

**Figure 6:**
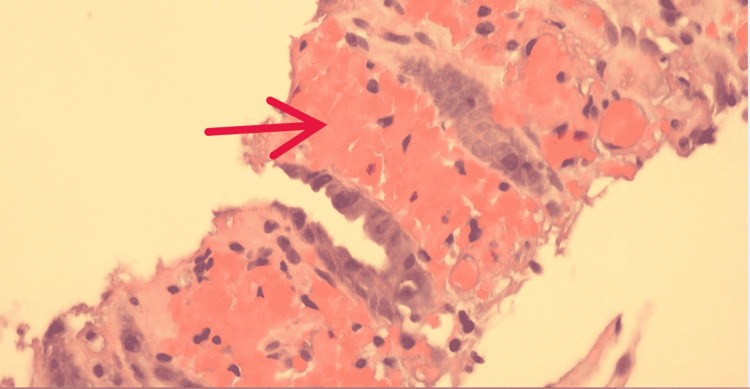
Colon biopsy, hematoxylin and eosin stain (50x) Red arrow pointing to the area of mucosal necrosis with mucosal hemorrhage, fibrin, and edema consistent with Ischemic colitis.

**Figure 7:**
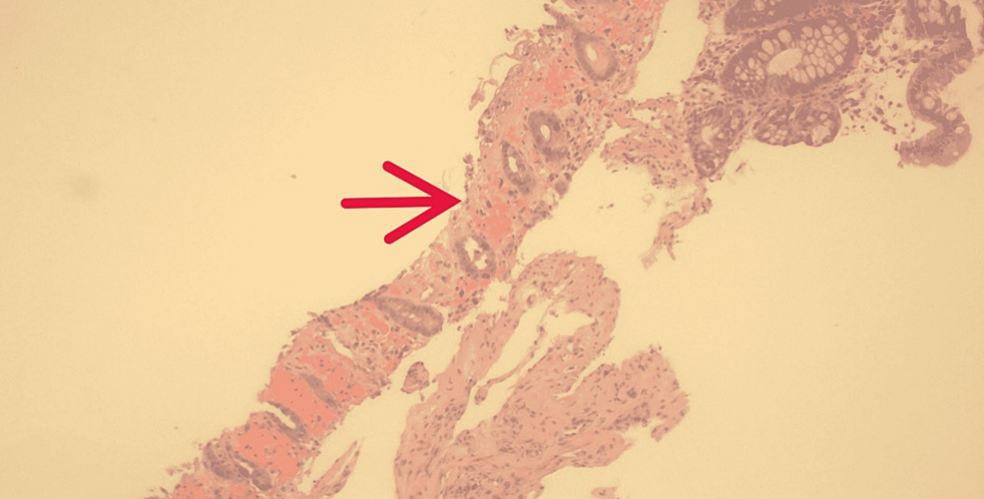
Colon biopsy, hematoxylin and eosin stain (10x) Red arrow showing markedly ulcerated mucosa with mucosal hemorrhage and fibrin consistent with ischemic colitis.

Histology showed hemorrhage with superficial erosion and ulceration consistent with ischemic colitis (Figures 6-7). The patient was discharged home on several laxatives to prevent constipation and recurrence of ischemic colitis. 

## Discussion

Ischemic colitis is an inflammatory condition that occurs when blood flow to the large intestine is reduced and inadequate to maintain cellular metabolic function. Ischemic colitis can affect any part of the colon, but the left colon is the most affected and accounts for 75% of cases of ischemic colitis. The splenic flexure is involved in about 25% of cases [3]. The splenic flexure and the sigmoid colon have limited collateral networks and are more prone to ischemic damage. Colonic ulceration and intestinal perforations secondary to stercoral colitis have been reported in the literature, but cases of stercoral colitis complicated by ischemic colitis are rare [2,4,5].

Stercoral colitis has an estimated postmortem incidence of 0.04% to 2.3% [2]. The pathogenesis of stercoral colitis results from stagnant hard fecal material resulting in decreased blood flow due to compression of the walls of the colon. This can further lead to ulceration, pressure necrosis, and eventually bowel perforation if the fecal impaction is not promptly diagnosed and treated [6]. Ischemic pressure necrosis occurs when colonic distention increases pressure above the bowel wall capillary perfusion pressure. The sigmoid colon and the rectosigmoid colon are the most common sites of fecal impaction; this is due to their narrower colonic diameter leading to higher intraluminal pressure. As a result, colonic ulceration is seen in 27% of cases of stercoral colitis, with 77% occurring in the rectosigmoid and sigmoid colon [7].

Features of computerized tomography scan of stercoral colitis include a dilated colon with thickening of the wall and stranding of the pericolic fat in an area that shows fecal impaction suggests ischemic colitis or wall edema [2]. Endoscopic findings of ischemic colitis include erythema, mucosal edema, erosions, or ulcers, including a single linear ulcer in the mesenteric border, as was seen in our patient during endoscopy. 

Treatment of stercoral colitis includes bowel regimen, fecal disimpaction, and enemas [8]. Surgical management, which includes surgical resection of the dilated colon, is indicated in patients with peritonitis secondary to colonic wall perforation. However, about 50% of uncomplicated stercoral colitis is managed conservatively without the need for surgical intervention [8]. Making a prompt diagnosis and initiating manual fecal disimpaction and an aggressive bowel regimen decreases complications of stercoral colitis and therefore reduces the morbidity and mortality associated with the disease [7].

## Conclusions

Stercoral colitis, complicated by ischemic colitis, has a high mortality and morbidity rate. Therefore, physicians should suspect this condition in patients with risk factors even with a limited or inconclusive imaging study. Our patient had a history of chronic constipation and had a limited CT abdomen and pelvis with intravenous and oral contrast; the pathology result from the colon biopsy was diagnostic for ischemic colitis. An even higher mortality rate occurs if stercoral colitis is complicated by colonic perforation and septic shock. It is, therefore, vital to avoid a delay in making an accurate diagnosis and initiating the appropriate treatment.
